# Labor management and neonatal outcomes in cardiotocography categories II and III (Review)

**DOI:** 10.3892/mi.2024.151

**Published:** 2024-04-01

**Authors:** Panagiotis Tsikouras, Efthimios Oikonomou, Anastasia Bothou, Dimimitrios Kyriakou, Theopi Nalbanti, Sotirios Andreou, Angelos Daniilidis, Panagiotis Peitsidis, Konstantinos Nikolettos, Georgios Iatrakis, Nikolaos Nikolettos

**Affiliations:** 1 Department of Obstetrics and Gynecology, Democritus University of Thrace, 68100 Alexandroupolis, Greece; 2 Midwifery Department of Neonatology, University Hospital Alexandra, 11528 Athens, Greece; 3 1st Department of Obstetrics and Gynecology, Papageorgiou Hospital, Aristotle University of Thessaloniki, 56429 Thessaloniki, Greece; 4 Department of Obstetrics and Gynecology, Helena Venizelou Maternity Hospital, 11521 Athens, Greece; 5 Midwifery Department, University of West Attica, 12243 Athens, Greece

**Keywords:** cardiotocography abnormalities, delivery management, neonatal outcome

## Abstract

The safe care of both mothers and fetuses during labor is a primary goal of all health professionals. The assessment of fetal oxygenation and well-being is a key aspect of perinatal care provided. Fetal heart rate (FHR) auscultation became part of daily obstetric practice in a number of countries during the 20th century and remains a key method of fetal monitoring, particularly in low-risk pregnancies. Cardiotocography (CTG) is the continuous monitoring and recording of the FHR and uterine myometrial activity, making it possible to assess the fetal condition. It therefore plays a critical role in the detection of fetal hypoxia during labor, a condition directly related to short- and long-term complications in the newborn. Herein, particular reference is made to the management of CTG category II and III standards, as well as to the handling of childbirth. In addition, specific FHR patterns are associated with immediate neonatal outcomes based on updated studies conducted worldwide. Finally, the prognostic significance of CTG and its potential as a prospective avenue for further investigation are also highlighted herein. Given that the misinterpretation of CTG findings is the most common cause of medical-legal responsibility, this knowledge field requires more emphasis and attention. The aim of the present review was to further deepen the knowledge on issues that mainly concern the safety and monitoring of pregnant women and fetuses during childbirth.

## 1. Introduction

It is possible to determine the perinatal state of the fetus by examining changes in the amplitude and frequency of the fetal heart rate (FHR). The continuous monitoring of the heart rate using cardiotocography (CTG) during labor is used to determine the adequacy of the fetoplacental unit (1). This method is applied to the vast majority of births that takes place in organized obstetric centers. The main aim of CTG is to assess the degree of adequacy of the fetal-placental unit and the early detection of changes in the FHR indicative of fetal hypoxia, as well as the timely taking of those actions to prevent unwanted events (1-3).

It is considered particularly important to correctly assess the recordings of the fetal rhythm during labor and to accurately diagnose possible deviations from the normal, as well as to investigate the possible causes. However, previous studies comparing continuous monitoring with the intermittent listening of fetal heartbeats have not demonstrated a significant improvement in reducing perinatal morbidity (4-9). In the present review, the international literature on the analysis and interpretation of CTG data is presented and reviewed, with specific reference to the management of class II and III FHR patterns, as well as to the management of labor and the association with the neonate in the immediate and distant future (4-9). The aforementioned studies also showed a significant increase in the frequency of obstetric operations, particularly caesarean sections. Nevertheless, despite the apparent lack of adequacy according to the aforementioned studies, clinical confidence in the method has not decreased. This fact is attributed by some either to the reluctance of clinicians to abandon the view that CTG is able to detect early fetal distress or due to the fact that the above views are the negative findings of well-designed studies.

## 2. Description of search strategy

The exploration of pertinent literature for the present review encompassed an extensive search across five distinguished databases, namely Google Scholar, Web of Science, Scopus, PubMed and Science Direct. This meticulous approach was undertaken to identify publications that would substantially contribute to the contemporary discourse on labor monitoring.

In the present review, two researchers, working independently, executed comprehensive searches utilizing a nuanced combination of keywords, including ‘cardiotocography’, ‘cardiotocography abnormalities’, ‘perinatal outcome’, ‘neonatal outcome’ and ‘delivery management’. The intention was to cast a wide net, capturing a diverse range of studies and scholarly works that delve into the multifaceted aspects of these topics.

Furthermore, an in-depth examination of the references within the initially retrieved articles was conducted. This supplementary step aimed to ensure a comprehensive and exhaustive inclusion of relevant research publications, enriching the overall depth and breadth of the review.

Upon the identification of the initial set of articles, a judicious and independent selection process ensued. The authors scrutinized the titles and abstracts with precision, employing rigorous criteria to exclude studies that deviated from the specific focus and parameters set for the present review. This stringent curation process was crucial in maintaining the scholarly integrity of the review, ensuring that only studies directly aligned with the scope were incorporated.

## 3. Categorization of cardiotocographic patterns during labor

CTG during labor is considered to be able to detect fetal hypoxia and/or acidosis, thus allowing for an early intervention and reducing adverse neonatal outcomes, such as cerebral palsy (CP). This is based on the theory that hypoxia during labor can affect the function of the central nervous system (CNS), which in turn affects the function of the FHR.

CTG parameters, including FHR baseline and variability appear to be independent prognostic markers of fetal acidosis and are associated with a significant reduction in early neonatal morbidity and mortality rates. Currently, to the best of our knowledge, there is no consensus among scientists regarding the sensitivity and specificity of CTG in predicting embryonic acidosis (10,11). The correct interpretation of CTG findings is essential for the implementation of a correct delivery management policy. In 2008, the US National Institute of Child Health and Human Development (NICHD) proposed a unified system for objective interpretation of recordings. The NICHD guidelines include six evaluation parameters: i) Contractions; ii) FHR variability; iii) accelerations; iv) (periodic or episodic prolonged) decelerations; tachycardia; and vi) bradycardia (12). The International Federation of Gynecology and Obstetrics (FIGO) and the National Institute for Health and Care Excellence (NICE) according to the revised guidelines they issued in 2015 and 2017, respectively, defined three categories of FHR CTG patterns, which are summarized below (10,13,14).

### Type I recordings (normal)

This includes an FHR of 110-160 beats/min, moderate variability (6-25 beats/1 min) and the absence of repeated decelerations (decelerations present in >50% of fetal contractions). This involves interpretation is in the absence of hypoxia/acidosis. In this case, no intervention is required apart from the improvement of fetal oxygenation.

### Type II recordings (undetermined significance/suspicious, 80% of recordings)

This includes the absence of at least one category I characteristic, but without pathological features, tachycardia or bradycardia not accompanied by the absence of variability, reduced or eliminated volatility without the presence of late decelerations, the absence of accelerations (particularly following stimulation). This also involves periodic or episodic decelerations including variable decelerations, prolonged decelerations, recurrent late decelerations type 1 (with moderate variability) variable decelerations with slow return to normal rhythm and jumpy accelerations. The interpretation in this case is a low probability of hypoxia/acidosis. Intervention involves the correction of reversible causes if identified or fetal blood sampling (FBS) to assess fetal oxygenation.

### Type III recordings (pathological)

This includes the absence of variability with repeated late decelerations (type 2), or variable decelerations, bradycardia, atrial rhythm (<100 beats/min), increased or decreased variability or sinusoidal recording, recurrent late or prolonged decelerations lasting >30 min or 20 min if variability is reduced or prolonged deceleration >5 min. The interpretation in this case is a high probability of hypoxia/acidosis. Immediate intervention is required in this case to correct reversible causes. Additional methods are required to assess oxygenation or if delivery is not possible in acute conditions, such as umbilical cord prolapse uterine rupture or placental abruption immediate delivery (14).

## 4. Interpretation of cardiotocographic data (NICE)

A normal condition includes an FHR ranging from 110 to 160 beats per minute, variability spanning 5 to 25 beats per minute, and an absence of decelerations. In the absence of concerning features, decelerations may manifest as early or variable, lasting for <90 min, with a duration >60 sec. Furthermore, reduced variability within the deceleration and a delayed return of the baseline FHR in the monitor post-deceleration are indicative of variable decelerations. A biphasic pattern and the absence of a shoulder point also contribute to the characterization of normality. These combined criteria collectively categorize the overall status as normal/reassuring (15-21).

Non-reassuring CTG encompasses an FHR falling within the range of 100 to 109 beats per minute and 161 to 180 beats per minute. Additionally, characteristics indicative of a non-reassuring status include a variability <5 beats per min persisting for 30 to 50 min or exceeding 25 beats per min for 15 to 25 min.

Furthermore, an altered state without alarming features lasting >90 min, or altered with an alarming feature present in <50% of contractions for >30 min, or altered with an alarming feature in >50% of contractions for <30 min contribute to the categorization of non-reassuring conditions (15-21).

Late decelerations occurring in >50% of contractions for <30 min without any other risk factor are considered non-reassuring. The presence of two physiques in an non-reassuring state also designates the condition as non-reassuring (15-21).

For a comprehensive assessment of the overall clinical picture, it is essential to investigate potential causes, monitor the fetal response and consider conservative measures such as repositioning, monitoring uterine contractions, administering intravenous fluids, paracetamol and antibiotics (15-21).

An abnormal CTG involves an FHR falling <100 bpm or exceeding 180 bpm. Additionally, features such as a variability <5 beats per min and persisting for >50 min, or exceeding 25 beats per min for >25 min, or a sinusoidal recording are indicative of abnormalities.

Furthermore, altered decelerations with concerning features present in >50% of contractions for 30 min (or less in the presence of risk factors), late decelerations persisting for 30 min (or less in the presence of risk factors), acute bradycardia, or a single prolonged deceleration lasting for >3 min contribute to the categorization of abnormal conditions (15-21).

The presence of one pathological or two non-reassuring traits designates the condition as pathological. In managing such cases, an escalated fetal response, conservative measures and further intervention, including FBS or delivery, depend on the evolving clinical scenario. In cases where prolonged bradycardia persists, the acceleration of labor may be considered, with a reassessment if recovery occurs within 9 min (15-21).

The concerning features of variable decelerations are defined as: A duration >60 sec, decreased within-deceleration variability, delayed return of the FHR to baseline following deceleration and a biphasic pattern (15-21).

## 5. Clinical decision making and interventions

Assessing, interpreting and evaluating CTG information requires multidimensional clinical skills that develop over time. The choice of clinical interventions must be based on the knowledge of physiology, maternal and fetal risk factors and clinical examination. The use of drugs, the administration of oxytocin, the use of epidural anesthesia, maternal pyrexia (infection), bleeding, the presence of meconium in the amniotic fluid, and finally, the stage and progress of labor are taken into account in formulating a birth plan (22-27).

Clinicians should provide a person-centered plan of care consistent with current guidelines for the assessment of maternal and fetal status during labor, as well as information obtained from the CTG. When fetal hypoxia/acidosis is suspected and intervention is required to avoid an adverse neonatal outcome, this does not necessarily suggest that immediate delivery by caesarean section or forceps should be performed. The underlying cause precipitating the suspicious or abnormal patterns (mostly tachypnea and maternal hypotension) can often be identified and treated with the subsequent restoration of adequate fetal oxygenation and return to normal recordings (22-27).

The term ‘intrauterine resuscitation’ describes a set of procedures meant to meet the needs of childbirth, advance the process, optimize the flow of blood via the uterus and the umbilical cord, raise the oxygenation level of the fetus and preserve sufficient activity of the myometrial tissue. These interventions improve maternal blood supply, placental perfusion and fetal oxygenation, and include the following: i) Changing the mother's position; ii) decreased myometrial activity; iii) the administration of fluids intravenously; iv) the correction of maternal hypotension; v) providing oxygen to the mother; vi) the diversification of maternal pushing efforts.

While research has shown a positive effect on fetal oxygenation from certain intrauterine resuscitation techniques, no evidence is yet available to indicate that they are capable of reversing fetal hypoxemia (27-31).

### Changing the mother's position

The lateral position relieves pressure on the maternal inferior vena cava and aorta, thereby maximizing cardiac output and return and thereby improving blood perfusion to the uterus. Lateral positioning (or repositioning) of the epitome can also relieve compression of the umbilical cord by altering the association between the uterine wall, umbilical cord and fetal body parts. Improved fetal oxygenation has been found in the left lateral position compared with the right lateral and supine positions. In addition, the lateral position has been associated with fewer late decelerations and more accelerations of FHR compared to the supine position (27-31).

### Decreased myometrial activity

As already mentioned, normal uterine contractions during labor cause the spiral arteries to constrict when the intrauterine pressure exceeds that of the spiral arteries. This causes a temporary interruption in the blood and oxygen supply to the fetus. Most fetuses can cope with reduced oxygen, provided that uteroplacental disposition and oxygen exchange are normal. Conversely, excessive uterine activity puts the fetus at risk of hypoxemia. In fact, some researchers have reported that persistent tachypnea during childbirth is closely related to an adverse neonatal outcome. Other characteristics of contractions, such as intensity, duration and resting tone are clinically important and should be included in the assessment of uterine activity (27-31).

Tachysystole has been shown to be associated with adverse maternal and neonatal outcomes, including an increased rate of caesarean section, vaginal delivery using fetal incision, admission to the neonatal intensive care unit and sepsis. Marked variability has been shown to be associated with reduced fetal oxygen saturation, with an increased incidence of absent or minimal variability with late and recurrent decelerations and finally fetal acidemia (27-31).

As even brief periods of marked myometrial activity affect fetal oxygenation, interventions should not be delayed until class II CTG patterns are present. Intrauterine resuscitation to reduce myometrial activity includes repositioning the epicenter to the left lateral decubitus position, fluid administration intravenously and, if oxytocin is administered, reducing or stopping the infusion (27-31).

Fetal acidosis should be ruled out when, in the presence of tachypnea, the FHR exhibits moderate variability and accelerations. In this case, it is possible to administer a tocolytic agent, such as terbutaline (27-31).

### Administration of fluids intravenously

The use of intravenous fluids in labor are considered to improve placental perfusion by maintaining or restoring maternal intravascular volume, although evidence of its effectiveness is limited. However, the administration of 500-1,000 ml Ringer's lactate solution has been found to significantly increase fetal oxygenation with the greatest increase observed in littermates receiving 1,000 ml of the said solution (27-31).

When recurrent late decelerations, prolonged decelerations, fetal bradycardia, or minimal or absent FHR variability are observed, the American College of Obstetricians and Gynecologists (ACOG) recommends the use of intravenous fluids to enhance uteroplacental blood supply and fetal oxygenation (32).

When intravenous fluids are administered, the close monitoring of fluid intake and output is required. This is particularly important when drugs that have been known to affect maternal fluid balance or hemodynamic stability have been used, as well as when certain medical conditions, such as cardiovascular disease, preeclampsia, or the administration of magnesium or other tocolytics are present.

Pregnancy is a risk factor for pulmonary edema due to a decrease in maternal colloid osmotic pressure, while increasing cardiac output and plasma volume. The intravenous infusion of large volumes of glucose-containing fluids should be avoided due to potential maternal and fetal complications (28-31,33).

The restoration of CTG patterns, following the administration of intravenous fluids, is indicative of an increase in uteroplacental diffusion and maternal oxygenation and consequently may allow vaginal delivery, provided that the delivery progresses at a rapid pace.

### Correction of maternal hypotension

Dehydration, the loss of a large amount of fluids, the supine position due to compression of the inferior vena cava, decreased venous return and cardiac output, as well as regional anesthesia predispose to transient episodes of hypotension. As a result, the perfusion of the uterus and the oxygenation of the fetus are limited. Hydration and placement in the lateral or Trendelenburg position usually restore blood pressure. Less often, drugs such as ephedrine are required, which have no known adverse effects on the fetus.

### Providing oxygen to the mother

Several studies have demonstrated an improvement in fetal oxygenation by placing the epitome in the lateral position, administering intravenous fluids, and administering oxygen via a non-rebreather mask set at 10 liters/min for 15-30 min. As there is insufficient evidence to address the ideal duration of oxygen administration, its use should be limited to the shortest possible duration necessary to achieve the desired effect. Oxygen administration to mothers without any evidence of maternal hypoxia to restore CTG class II or III patterns has been disputed (33).

Nevertheless, the majority of scientists support the prudent and gradual administration of oxygen at high rates in specific cases. Recurrent late or prolonged decelerations, significant variable decelerations, bradycardia, or persistent minimal or absent variability may be reversed by the administration of maternal oxygen. The administration of oxygen in the antenatal period should follow the application of other intrauterine resuscitation measures (recumbent positioning, interruption or reduction of administered oxytocin, and hydration) due to the potential damage it may cause to maternal and fetal tissues (32,34,35).

### Diversification of maternal pushing efforts

During the second stage of labor, maternal pushing efforts may be associated with FHR decelerations. Suggested remedial approaches include glottis-open exhalation rather than Valsalva exhalation, making fewer exhalative efforts with each contraction, shortening each exhalative effort, exhaling every second or third contraction, and, in terms of rates that have subject to regional anesthesia, the extrusion only when the need is perceived. Lewis and Downe (9), as well as others, recommended pausing expulsive efforts until the recovery of decelerations and the return to pre-expulsion normal baseline and variability (36).

## 6. Newborn outcomes

One of the greatest challenges in obstetrics is confirming the right time for the birth of the fetus. During labor, unnecessary interventions may cause maternal harm, while delayed interventions may result in fetal or neonatal death or permanent CNS damage. The time required to complete the delivery in order to prevent an adverse neonatal outcome is unknown. There are reports that neonatal encephalopathy (NE) occurs in 3‰ of all full-term live births and is a major predictor of later neurodevelopmental disorder. In 15-20% of cases it will lead to neonatal death, while 25% of affected newborns will suffer permanent neurological damage. NE is a clinical syndrome, which is manifested by a reduced level of consciousness, seizures, respiratory failure and/or reduced tone and reflexes (1,37-40).

It is estimated that 70% of cases of NE are the result of events that occurred prior to the onset of labor, while <10% result from postnatal complications, such as severe respiratory distress, sepsis and shock. The majority of the remaining cases are due to hypoxia/acidosis during labor, although their incidence varies depending on the care provided. NE can also be caused by hypoxia/acidosis occurring before delivery or during the postnatal period (1,37-40).

Hypoxia/acidosis during labor can result in acute neurological impairment known as hypoxic ischemic encephalopathy (HIE). An estimated 1.5 cases occur for every 1,000 live births. NE can have a variety of non-hypoxic causes, therefore confirming metabolic acidosis in the cord blood or neonatal circulation in the first few minutes of birth is necessary for diagnosis. Low Apgar scores at 5 and 10 min of life are also critical, as is early imaging evidence of brain edema.

Multisystem organ damage may coexist, involving the digestive, urinary, cardiovascular, circulatory, endocrine and gastrointestinal systems, although the severity of neurological damage is not necessarily associated with this.

The occurrence of a clinical event (prolonged maternal hypotension, uterine rupture, umbilical cord prolapse, etc.) during labor may indicate whether the cause of hypoxia/acidosis pre-existed or occurred during labor. However, previous studies have concluded that clinical events alone are poor prognostic indicators for NE (41-46).

Continuous CTG monitoring from the onset of labor may also help to determine the timing of the lesion. Reduced variability and the absence of accelerations from the onset of labor indicate a fetus with pre-existing damage.

CP is the most common form of disability in childhood and consists of a heterogeneous group of non-progressive motor and static disorders, often accompanied by cognitive and sensory disorders, epilepsy, nutritional deficiencies and secondary musculoskeletal alterations. The incidence of CP worldwide has remained constant at 2-3 per 1,000 live births for over four decades, despite significant improvements in obstetric and neonatal care provided (23,46-48).

Although in developing countries ~50% of cases are associated with prematurity, in developed countries, the rate of NE appears to decrease among preterm infants, but remains stable among full-term infants. Full-term neonates account for 50-60% of cases and tend to be the most severely affected.

Spastic tetraplegia and dyskinetic type CP are late neurological complications strongly associated with hypoxia/acidosis during delivery. One of the most severe types of CP that impairs the ability to relax the muscles in the upper and lower limbs is spastic quadriplegia. It is described in almost 90% of cases of children with a very low birth weight resulting from an intermittent decrease in fetal oxygenation that occurs over a period of at least 1 h (23,46-49).

Dyskinetic CP occurs mostly in full-term neonates and originates from an acute and significant decrease in fetal oxygenation, as occurs in the case of umbilical cord prolapse, placental abruption, uterine rupture and maternal cardiovascular shock. CP mainly in its most severe forms, is often accompanied by neurological disorders, namely mental retardation in ~50% of cases, epilepsy in 25-45%, speech disorders in 40%, visual impairment in 40% and hearing impairment in 10-20% of cases. Neurodevelopmental disorders are common, while characteristic disorders of the autism spectrum may occur in >7% of cases. Other systems that may be affected are the reproductive, urinary, gastrointestinal, endocrine and musculoskeletal systems, while chronic pulmonary disease will eventually lead to death (23,46-49).

The etiology of CP is complex and includes a wide range of risk factors before, during and after delivery. Prematurity, low birth weight, hypoxia/acidosis during delivery, chorioamnionitis, CNS lesions, fever during delivery, multiple pregnancy, coagulation disorders, ischemia, maternal thyroid disease, obesity and placental pathology are the most commonly reported risk factors (23,46-49).

Hypoxia/acidosis during labor is probably the most extensively studied risk factor accounting for only 10-15% of cases of NE. According to the ACOG and American Academy of Pediatrics (AAP), hypoxia during labor can be considered responsible for the development of CP when the following apply (50,51): The presence of metabolic acidosis documented by cord blood gas measurement (pH <7.0, base deficit ≥16 mmol/l or lactic acid ≥10 mmol/l) within 1 h of delivery. The early onset of severe or moderate neonatal CP can occur in children born after 34 weeks of gestation.

A neurological examination within the first 24 h following delivery will reveal abnormal findings from ischemic brain damage caused during delivery. The outcome is proportional to the severity of the damage, i.e., the heavier the damage, the more unfavorable the outcome.

A number of events during labor have been statistically associated with CP, including vaginal delivery with fetal ulcer, cesarean section and breech delivery. Other associated factors are the presence of meconium, mainly thick in the amniotic fluid, the aspiration of meconium, severe damage to the placental vessels, placental abruption and prolapse of the umbilical cord.

Additionally, in a previous study, MacLennan *et al* (48) found no association between elective caesarean section and CP. By contrast, an increased risk of developing CP was identified as a consequence of the necessity for an emergency caesarean section. Neonatal risk factors associated with CP include a low Apgar score at 5 min, epilepsy, respiratory distress syndrome, hypoglycemia, jaundice, hyperlipidemia, neonatal sepsis and meningitis, as documented in reported cases (23,46-51).

## 7. Value of cardiotocography in pregnancy and prospects

For over half a century, CTG has been introduced into daily obstetric practice as a method of simultaneous monitoring of myometrial activity and FHR, both during pregnancy and during the progress of labor. The initial enthusiasm for the use of the method in high- and low-risk pregnancies was followed by the concern raised by subsequent investigations, regarding its prognostic value for metabolic acidosis and adverse neonatal outcome (51-54).

Although there is a clear association between pathological CTG and the condition of the newborn, as confirmed by the Apgar score, the presence of acidosis, HIE and the subsequent disorders in neuromotor development, the recordings often have a high false-positive or false-negative predictive value. This means that pathological recordings, even type III recordings (pathological), do not necessarily indicate neonatal morbidity or mortality, while normal recordings may be observed in cases of fetuses with adverse clinical outcomes (51-56).

According to FIGO, an umbilical artery pH <7 or base deficit ≥12-16 mmol/l constitute states of metabolic acidosis used to determine whether CTG interpretation can accurately predict fetal asphyxia (51-56).

Recent research has concluded that specific patterns of FHR during labor are associated with metabolic acidosis and neurological damage. Graham *et al* (45) reported that absent or low variability (range <5 bpm) for at least 1 or 2 h prior to delivery, alone or in combination with late decelerations and the absence of accelerations, are the most critical CTG parameters for predicting severe acidosis. These conclusions were confirmed in other research (48).

Several other scientists have further emphasized the prognostic value of variable decelerations and tachycardia (50-56). According to Vintzileos and Smulian (34), any deceleration that causes tachycardia is critical, while frequent episodes of tachycardia or continuous tachycardia are the first signs of fetal danger. In 2018, Cahill *et al* (57), in a prospective clinical study with a sample size of 8,580 subjects, concluded that bradycardia <100 bpm, tachycardia >180 bpm, late and prolonged decelerations were strongly associated with a low umbilical artery pH (51-56).

In addition, for the prognosis of fetal acidemia, Tsikouras *et al* (58) pointed out the value of the total area of deceleration (depth by duration), as well as the tachycardia lasting 10 min, even in the presence of moderate variability. Frey *et al* (23) agreed with these findings and further reported that in >80% of these cases, there is fetal asphyxia.

Research has also confirmed earlier conclusions that type II records or type III recordings (pathological) in the 2nd stage of labor are an independent indicator of metabolic acidosis. However, Clark *et al* (25,26) concluded that, of the neonates born with metabolic acidosis, even under ideal conditions, only 50% could be identified to hasten delivery. This also determines the limits of the CTG method in monitoring the fetus during labor (29,55-60).

## 8. Summary and prospects

The contractions of the myometrium during labor compress the protruding part of the fetus and the umbilical cord, resulting in the manifestation of marked changes in the intrauterine environment. The adrenal glands release catecholamines in reaction to stress on the developing fetus. The fetal ability to deal with the stress caused by contractions and the pressure of the umbilical cord stress depends on the physiological reserve of the fetus, as well as on the possible presence of infection or meconium-stained amniotic fluid. Fetal hypoxia is not the only damaging factor (61-63). There are indications that the fetal response to infection/hyperpyrexia may be a cause of fetal CNS damage. Fetal cardiac function is controlled by the autonomic nervous system with minimal influence from the somatic nervous system. Fluctuations between the parasympathetic and sympathetic systems determine the basic variability of the FHR. The somatic nervous system causes the accelerations that result from CNS action to control of fetal movements. The action of the sympathetic system is essential for fetal survival and causes an increase in FHR. Conversely, the parasympathetic decreases the fetal heart rate as a result of stimulation of chemoreceptors of the carotid sinus and aortic arch and baroreceptors located peripherally in the carotid bulb and aorta and centrally in the brain. The stimulation of baroreceptors is caused by umbilical cord and head pressure and contributes to the reduction of FHR recorded as the appearance of short duration variable decelerations (63-66). Chemoreceptors are stimulated by the increase in hydrogen ions, the accumulation of CO_2_ and the decrease in the partial pressure of oxygen.

The increase in catecholamine secretion is a reaction product to impending fetal distress and manifests as a slowly evolving increase in FHR as a result of vasoconstriction and redistribution of blood flow to vital organs. Reduced uteroplacental reserve is a consequence of pregnancies complicated by intrauterine growth retardation, prematurity or extension. The term hypoxemia describes the condition in which there is a decrease in blood supply at the level of the placenta and umbilical cord and a decrease in oxygenation in the peripheral circulation of the fetus. This condition is often observed during normal childbirth, it is a result of uterine contractions and most fetuses cope adequately for a long period of time with the occurrence of such episodes without the establishment of damage. The term hypoxia describes the condition where there is an interruption of blood supply for longer periods of time, resulting in the reduction of oxygenation of peripheral tissues and organs of the fetus. When hypoxia persists for more extended periods of time, the fetus activates anaerobic metabolism that leads to the establishment of metabolic acidosis. In fetuses with a reduced reserve, such as intrauterine growth retardation, or prematurity or infection, the establishment of metabolic acidosis occurs earlier. The term asphyxia describes the extreme adverse case in which there is a major worsening of the prolonged interruption of reduced oxygenation, leading to the inability to respond to compensatory mechanisms and the establishment of a combination of metabolic acidosis and hypoxia (63-67). This leads to significant damage to vital organs, such as the CNS and possibly, intrauterine death. Long-term or chronic hypoxia occurs due to a reduction in placental blood flow for a long period of time, associated with chronic pathological conditions such as pre-eclampsia or intrauterine growth retardation. Before delivery, the fetus copes for a long period of time by redistributing blood flow to vital organs with a slowdown of intrauterine growth rate and by controlling the increase in lactic acid levels.

During labor, gradually developing hypoxia is identified by a significant reduction or abolition of variability and the appearance of repeated late decelerations. The presence of such a severe hypoxic attack causes the fetal reaction, which is initially expressed by the appearance of either variable decelerations (due to compression of the umbilical cord) or the appearance of late decelerations (due to placental insufficiency), the disappearance of the accelerations (result of an effort to save energy), the gradual increase in FHR due to hypoxia and increase in catecholamine secretion in deepening and increase in the range of decelerations (with increasing hypoxia of the myocardium) and finally, in a progressive abolition of variability (due to a greater lack of oxygen resulting in suppression of the autonomic nervous system). Acute hypoxia is characterized by a sudden decrease in placental/umbilical blood flow and develops within a short period of time (minutes). The causes of this include acute events, such as placental abruption, hypertonia or uterine rupture. Recordings show prolonged decelerations progressing to bradycardia (60-63).

Immediate treatment is required by arranging delivery or eliminating hypertonia. Subacute hypoxia occurs due to the recurrent compression of the umbilical cord. It worsens in cases of oligoamnios or the prolongation of pregnancy, particularly in the second stage of labor. The fact that hypoxic attacks have a slow progression at the beginning and are persistent after a sufficient period of time allows for the gradual adaptation of the fetus. These adaptive actions begin to lose their effectiveness when severe metabolic acidosis is established (pH ≤7) with suppression of myocardial function, reduction of vascular tone and establishment of hypotension, and ischemic brain damage (63-67).

The studied characteristics of FHR are divided into basic and periodic-episodic. The basic characteristics are those recorded in the intervals between uterine contractions and include the FHR and variability. The periodic characteristics are those recorded in relation to uterine rhythms, while the corresponding episodes include changes not obviously related to myometrial activity. The evaluation of the fetal condition during pregnancy and delivery consists of the analysis and interpretation of the FHR. The analysis refers to the definition of the characteristic patterns of the CTG sample, while the interpretation refers to their clinical significance.

The correct evaluation of a CTG, particularly in full-term pregnancies, requires the knowledge that the regulation of FHR depends on the degree of fetal maturity based on the gestational age. In particular, the basic FHR gradually decreases from the 28th week of pregnancy until the end of it by approximately one pulse per week, the variability is greater after the 28th week, the frequency of FHR accelerations increases after the 24th week, while decelerations decrease.

It is particularly important that under normal conditions, the fetus *in utero* exhibits a clinical alternation of phases of rest and movement, the duration of which varies, depending on the gestational age (in full-term fetuses it can reach up to 70 min). Depending on the phase of the motor activity of the fetus, cyclic changes in variability and basal FHR occur, respectively (63-67).

The settings required for the operation of the CTG to follow international standards are the following: Paper speed at 1 cm/min, sensitivity at 20 bpm/cm, and the FHR range drawn on the paper between 50 and 210 bpm. The recording, as usual, is carried out for at least 20 min with a signal reception frequency of 4 Hz. The evaluation of CTG recordings should be done in relation to the clinical picture, the gestational age, the state of the mother's health, the previous results regarding the fetal condition, the medication and any therapeutic intervention that requires the previous mother-fetal evaluation.

A complete description of the FHR sample should include the reporting of risk factors by the mother, as well as the quantitative and qualitative description of both myometrial activity (frequency, duration, intensity of contractions and resting tones) and FHR (baseline FHR, volatility, accelerations, decelerations) (65-69).

As regards myometrial activity, contractions are quantified as the number of contractions in a 10-min period as a result of the average of 30 min of recording. FHR is defined as the average value of FHR showing a variation of up to 5 pulses/1 min in a monitoring interval of 10 min with the exception of episodic or periodic changes of periods of marked variability, and those parts of FHR showing a difference of more than 25 beats/1 min.

During each 10-min monitoring interval, it is imperative that the FHR persists for a minimum duration of 2 min. Failure to meet this criterion necessitates a return to the preceding 10-min monitoring interval for the accurate determination of the FHR. In current clinical practice, the FHR ranges between 110-160 beats/min. Values <160 represent bradycardia and those >160 define fetal tachycardia. FHR variability is the anomaly observed in the vast majority of cases in the FHR plot line and represents slight temporal differences during the pulse-to-pulse calculation of the FHR (63-69).

Basic FHR volatility represents FHR fluctuations of the order of two cycles/1 min or more. These fluctuations are somewhat similar to sinus waves, which unlike the sinus rhythm, are non-uniform in amplitude and frequency. The above definition is considered sufficient for clinical observation and interpretation although two forms of variability have been recognized (63-69).

The first or short interval variability or beat-to-beat variability defines the beat-to-beat variability that arises from the fact that there are very small differences in the duration of the intervals between the R beats in the normal fetal electrocardiogram. The second is the long-term variability which is described either as a range deviation or as a frequency of long-term indirect changes of the FHR covering a time cycle of at most 2 min. However, for a number of clinicians, variability is a single clinical entity. The origin of FHR variability is obviously a complex process with the contribution of various cyclic phenomena, such as respiratory arrhythmia, blood pressure fluctuations and the process of temperature regulation. The cerebral cortex, the midbrain and the vagus nerve work together to determine the variability and the cardiac excitatory system.

Periodic changes in FHR include those associated with uterine contractions, such as late decelerations, early decelerations, variable decelerations, sustained decelerations and accelerations. The latter exhibit a close association with the physiological variability of FHR and consequently have a positive predictive value regarding normal fetal oxygenation. Therefore, it is important to separate them from the presence of normal complexes of long-term variability. The assessment of the severity of the decelerations is based on the determination of the lowest value of pulses/1 min (nadir) simultaneously excluding the instantaneous spikes and electronic artifacts. The duration is calculated from the beginning to the end of the alteration in min or sec. A similar assessment applies to accelerations. Decelerations are characterized as recurrent-persistent when they accompany 50% of uterine contractions in each 20-min monitoring interval (63-69).

Recordings are considered normal when the FHR ranges between 110-160 beats/1 min, there is short-term variability and the corresponding long-term variability ranges between 6-25 beats/1 min. It has been widely clinically accepted that the positive predictive value of the above normal recordings it does not apply to cases where either a traumatic delivery will follow or the fetus suffers from some congenital disease.

During the follow-up, it is possible to record certain elements which, although different from what is considered normal, are not necessarily pathological. The above elements are characterized as miscellaneous and include modifications to the characteristics of the FHR, the variability and the periodic elements (63-69).

In detail, the above deviations include the following: i) Bradycardia, which is the initial response of a healthy fetus to acute hypoxia or suffocation. There are a number of causes of bradycardia unrelated to asphyxia, such as hypothermia, cardiac bradyarrhythmias such as complete atrioventricular block, and certain medications such as β-adrenergic blockers and xylocaine. However, usually, the finding of bradycardia indicates either the gradual drop in fetal oxygenation, which is a consequence of maternal apnea or amniotic fluid embolism, or the reduction of blood flow in the umbilical vessels such as following the prolapse of the umbilical cord, or the reduction of blood flow in the uterus vessels such as after severe maternal hypotension. ii) Tachycardia which can be observed in some cases of asphyxiation, but always accompanied by other alterations in the recordings. Tachycardia accompanied by normal variability and the lack of alterations occurs due to causes other than hypoxia. Causes of tachycardia not associated with asphyxia are mainly caused by maternal or fetal infection and particularly, chorioamnionitis as well as sympathomimetic and parasympatholytic drugs. iii) Reduced variability: The presence of physiological variability is perhaps the most critical indicator of a good perinatal condition of the embryo-placental unit. The reduction of variability is combined by cerebral asphyxia and the failure of the hemodynamic compensatory mechanisms to act successfully. The finding of an apparent absence of variability without periodic changes may be due to: a) The rest-sleep status of the fetus; b) idiopathic causes; c) drugs that act on the CNS (narcotics); d) congenital or acquired CNS lesions; e) disorders of the excitatory system of the heart; and f) severe suffocation (63-69). iv) Late and early decelerations: There are two types of late decelerations, the first type reflexive late decelerations is observed when an acute episode (a sudden drop in blood flow due to maternal hypotension) affects a previously normally oxygenated fetus during labor. The second type is observed when there is insufficient oxygenation of the fetal brain and myocardium due to placental insufficiency (pre-eclampsia, intrauterine growth retardation, effect of prolonged asphyxiating stress) (66-71). The distinction between the aforementioned types is based on the presence of physiological variability in the first type. Early decelerations are a form of reflex late decelerations. v) The variable decelerations that are the response of the vagus either to compression of the umbilical cord or to significant compression of the fetal head as occurs in the ejection stage. Their severity is directly related to the presence or abnormal variability. vi) Accelerations that accompany contractions without particular prognostic value, but may be indicative of an active and healthy fetus. The clinical management of abnormal recordings of the FHR is specialized according to the predominant alteration and the degree of severity of the recordings. In the case of late decelerations, therapeutic intervention takes place only in the case of their persistent appearance. When these are accompanied by physiological variability, the treatment is basically based on increasing the blood flow to the fetoplacental unit by hydrating the patient, changing the position and administering oxygen, tocolytic drugs and ephedrine.

The main purpose of the treatment is to eliminate the decelerations from the recordings before they become prolonged and pronounced, and before a reduction in variability mainly occurs. According to clinical observations, the majority of embryos with normal development are able to compensate for such alterations for a period of ~30 min. This period of time allows comfortable preparation for possible obstetric surgery (65-69).

The second type of late decelerations with reduced or abolished variability in most cases characterizes either already asphyxiated fetuses or those that are about to become asphyxiated. The immediate termination of labor is absolutely indicated in these cases. However, it is possible in a small number of cases that there is no suffocation. These cases can be recognized either by the application of stimulation (inducing accelerations when the fetal scalp is touched to obtain blood), or by analyzing the blood gases of the fetus and detecting possible metabolic acidosis (65-71).

In the case of recording variable decelerations, the type of treatment depends basically on the time of their appearance. The treatment of the usually light-moderate decelerations that take place during the latent phase of labor consists of changing the position of the rate. If these persist and worsen to a heavy extent, it is recommended to perform an abdominal ultrasound to determine the adequacy of the amniotic fluid. If the amniotic index is reduced, it is possible to apply amniocentesis, although in most cases, a simple change of position is preferred, as it is less invasive (65-71).

The same method of treatment also applies to the variable decelerations of the first stage. If they continue to appear even after the position of the interest rate has been changed, it is sufficient to apply continuous cardiotocographic monitoring and intervention. The recording of heavy variable decelerations is a possible indication of future fetal asphyxia which in fetuses with normal development it takes ~30 min to install. In these cases, systematic and careful monitoring of the variability in the intervals between the contractions and immediate termination of labor before the complete abolition of variability is required, unless asphyxia is ruled out by the application of fetal blood gas analysis (67-71).

Severe prolonged variable decelerations and bradycardia may accompany prolonged contractions. In these cases, it is recommended to reduce the rate and dose of oxytocin administration, the application of the lateral position and possibly the administration of a tocolytic preparation.

The treatment of the varying decelerations of the 2nd stage of labor is somewhat different. In this case, a number of clinicians apply some method of invasive vaginal termination of labor (forceps or vacuum). It is also good to avoid pushing out the pregnant woman at every contraction.

As regards the treatment of bradycardia, the general opinion is that the degree of reduction of FHR is directly related to the possibility of development of fetal distress. An important role is played in this case by the variability of the FHR and the presence or non-persistent decelerations. The recording of physiological variability is an indication of sufficient compensation on the part of the fetus for an unlimited period of time, with the result that simple monitoring is sufficient even when the FHR varies between 80-100 pulses/1 min. However, when the FHR fluctuates at lower levels, it is recommended to take measures to increase it and prepare for the termination of labor (5,44,69-71).

The completion of labor should be done in an extremely short period of time when a reduction in variability is observed. The treatment of bradycardia in the 2nd stage of labor is modified by the possibility of immediate intervention for vaginal termination of labor. In this case, as long as the projecting part of the fetus is low, it is possible to treat the bradycardia conservatively with the premise of immediate, if necessary, vaginal delivery.

Recapitulating in clinical practice, the interpretation of FHR recordings is a constantly evolving dynamic process that takes into account a number of obstetric factors, specific alterations of FHR, and above all, the progressiveness of the development of the above elements. There are usually two scenarios of developments. The first includes the evolution of type 1 late decelerations into type 2 decelerations with the progressive increase in intensity, the subsequent decrease in intermediate variability, and the presence of finally persistent decelerations with elimination of variability (69-71). There are some exceptions to the above scenario, such as in cases of the prolongation of pregnancy or the establishment of chorioamnionitis where the reduction or abolition of variability may be combined with slight decelerations and relative tachycardia.

The second scenario involves the progressive worsening of mild and moderate variable decelerations with the descent of the projecting fate of the fetus. In this case, the clinician is usually not able to know whether the above recordings are due to compression of the head or a partial pressure occlusion of the umbilical vessels; thus, the degree of fetal hypoxia should also be assessed, particularly when there is also a reduction or eliminating volatility (69-71).

The presence of changing decelerations is usually indicative of adequate oxygenation of the fetal brain and therefore the decision to wait; conservative treatment or immediate intervention will be based on the presence and quality of the variability in combination with the assessment of the severity of the lesions.

In conclusion, the clinical future of continuous or non-CTG monitoring still remains unclear. In the authors' opinion, continuous CTG monitoring is recommended in high-risk pregnancies, as well as the application of the 20-min insertion test with or without a stimulation test, as well as periodic 5-min monitoring every 30 min in low-risk pregnancies (5,44,71-73). In this manner, the early detection of most cases of progressively established and developing fetal asphyxia becomes possible and immediate measures are taken before irreversible damage occurs. Finally, it should be emphasized that the monitor is recording uterine activity adequately and concurrently with the FHR and not maternal heart rate.

## 9. Conclusion and future perspectives

In recent years, in an effort to identify a more accurate method of predicting metabolic acidosis and adverse neonatal outcomes, in addition to electronic fetal monitoring, researchers have introduced the concept of the fetal reserve index (FRI). The FRI includes measurements of five components of CTG, namely FHR, variability, accelerations, decelerations and abnormal myometrial activity by combining them with the presence of medical, obstetric and fetal risk factors.

By introducing FRI as a parameter in the computerized CTG analysis, interpretation and scoring system, it has been concluded that this more reliably and earlier identifies fetuses who are at a risk of hypoxic and mechanical forces of labor than the very common type II recordings or type III recordings (pathological) which occurs very slowly (5,44,71-73).

FRI is a better indicator of discriminating urgent interventional delivery and adverse neonatal outcomes than traditional CTG, since the greater majority of neonates with cerebral palsy will not reach the point where intervention is required in type III recordings (pathological). However, additional investigation and validation are imperative to establish the quantitative approach, the FRI, as a potential replacement for the existing subjective interpretation of CTG. Other research developed an index of heart rate variability related to fetal parasympathetic activity, the fetal stress index (FSI). FSI is a non-invasive method of continuous analysis of fetal parasympathetic tone. Although further studies are needed for clinical validation, FSI could be an interesting method to assess fetal well-being (5,44,71-74).

A promising method is, according to Ugwumadu (75), the calculation of the mean arterial pressure of the fetus during hypoxia. Superior to pH measurement for confirmation or non-adverse neonatal outcome. Provided the necessary technology is available, it is a more ideal monitoring parameter than pH and lactic acid measurement (75).

Research in the field of electronic fetal monitoring in recent decades has aimed at improving the understanding of the FHR patterns most likely associated with fetal acidosis, but also those associated with obstetric emergencies. These FHR standards are relatively rare, particularly at maturity rates. When they do occur, however, immediate intervention to optimize fetal oxygenation and hasten labor has been shown to help prevent neurological damage or fetal death (73,74).

Numerous supplementary or alternative methodologies are currently in development or undergoing exploration (76). The Novii^®^ Wireless Patch System is an innovative maternal-fetal monitoring device that offers non-invasive measurement and display of the FHR, maternal heart rate and uterine activity. Unlike traditional methods, it detects fetal and maternal electrocardiogram (ECG) signals and uses electromyogram (EMG) signals from the uterine muscle for contraction monitoring. The system's wireless design provides comfort and flexibility during labor and childbirth, making it particularly beneficial for patients with a high body mass index and for those who desire frequent position changes. Overall, the Novii system represents a promising advancement in intrapartum monitoring technology (77,78).

The Moyo FHR monitor is a portable device designed to enable women to remain mobile during labor. It offers both intermittent and continuous monitoring options, and is lightweight and easy to transport. Equipped with a nine-crystal Doppler ultrasound sensor, it accurately measures and analyzes the FHR. The device displays FHR readings on its main unit screen and provides audible sound cues. Overall, the Moyo FHR monitor provides convenient and reliable monitoring of fetal well-being during labor (78). The Moyo FHR monitor allows for the review of the FHR tracing for up to 30 min at a time on the monitor. To differentiate between maternal heart rate and FHR, the Moyo main unit incorporates maternal heart rate electrodes for comparison. Through programmed analysis, the device generates alerts in green (normal), yellow (warning) and red (action) categories. Although not currently accessible in the USA, the manufacturer, Laerdal Global Health, is dedicated to providing the product on a not-for-profit basis to nations with the highest rates of maternal and neonatal mortality (78).

Current standards of fetal monitoring necessitate clinicians to interpret FHR tracings, while considering various external factors that may influence the pattern. Active research focuses on integrating smart computer systems capable of interpreting FHR patterns to alert clinicians about potential issues, with examples including the early Sonicaid system and programs, such as Local Diagnostics, Stan S31, PeriCalm and TraceVue. Although these automated systems aim to acquire, analyze, and prioritize vast data encountered during labor, their refinement, particularly through artificial intelligence and machine learning, is ongoing, and their definitive impact on improving pregnancy outcomes is yet to be established (79,80).

Through the utilization of sophisticated fetal monitoring systems, there is an optimistic outlook on the prospective advancement of fetal monitoring. The aspiration is for such systems to evolve to a degree where they can effectively diagnose and formulate a care strategy for both the patient and the fetus, particularly concerning acidemia, by analyzing FHR patterns alongside clinical input. The ability to accurately predict pregnancies predisposed to acidemia or requiring cesarean delivery holds the potential for early intervention, thereby mitigating complications, such as those associated with prolonged labor. Despite advancements achieved thus far, it is anticipated that considerable time will be required before these systems reach their full developmental potential (76).

In conclusion, the early recognition of patterns that signal the presence of potentially devastating acidosis is critical for the management of these conditions. The decision to induce labor depends on several factors, including the availability of human and material resources, the etiology of the decreased oxygenation, and the duration of an abnormal FHR pattern.

Standardization of terminology, multidisciplinary training in FHR interpretation and underlying physiology, and collaborative and teamwork-based management remain the safest approach to obstetric care delivery.

## Data Availability

Not applicable.
